# Application of Olive By-Products in Livestock with Emphasis on Small Ruminants: Implications on Rumen Function, Growth Performance, Milk and Meat Quality

**DOI:** 10.3390/ani11020531

**Published:** 2021-02-18

**Authors:** Ouranios Tzamaloukas, Marina C. Neofytou, Panagiotis E. Simitzis

**Affiliations:** 1 Department of Agricultural Sciences, Biotechnology and Food Science, Cyprus University of Technology, Limassol 3036, Cyprus; ouranios.tzamaloukas@cut.ac.cy (O.T.); maneofyt@gmail.com (M.C.N.); 2 Laboratory of Animal Breeding and Husbandry, Department of Animal Science, Agricultural University of Athens, Iera Odos, 11855 Athens, Greece

**Keywords:** olive by-products, ruminants, milk quality, monounsaturated fatty acids, meat quality

## Abstract

**Simple Summary:**

Olive oil is one of the main components in the Mediterranean diet that is known worldwide for its beneficial effects on human health due to its high content of monounsaturated fatty acids (MUFAs). During its extraction, a great quantity of olive by-products (OB) is generated that poses a risk to the environment due to its high organic load. Utilization of OB as a part of the ruminants’ diet could minimize the costs related to animal feeding and OB management and contribute to the preservation of natural resources. At the same time, their application in ruminants’ nutrition enables the sustainable use of high-added value bioactive ingredients inside food chains that improve milk and meat quality characteristics and fortify consumer health, without negative effects on rumen function, metabolism and productivity.

**Abstract:**

The olive oil industry has a leading position in the Mediterranean countries, resulting in the production of considerable quantities of the respective by-products (OB) that constitute an important environmental issue. OB contain valuable nutrients and bioactive components that can be re-used under the bioeconomy strategy, and several chemical, physical, and biological processes have been evaluated with the intention to improve their nutritional value. One feasible application of OB is their incorporation in the diets of livestock and especially ruminants due to their high fiber content. As indicated by numerous studies, OB dietary supplementation increases the levels of monounsaturated fatty acids (MUFAs) and decreases that of saturated fatty acids (SFAs) in the milk and meat of ruminants with beneficial effects for consumers’ health. At the same time, environmental impact and feeding costs are reduced without detrimental effects on ruminal fermentation, nutrients utilization, growth performance, carcass traits, milk yield and composition.

## 1. Introduction

The traditional Mediterranean diet has beneficial effects on the health status of populations in South Europe. One of its main components is olive oil, which provides a high monounsaturated to saturated fat ratio in the diet [1]. Cultivation of olive (*Olea europaea*) tree is strongly related to the economy of the Mediterranean countries. In 2014, Spain, Italy, and Greece produced more than 1.74, 0.30, and 0.21 million tons of olive oil, respectively [2]. However, olive oil extraction generates a considerable quantity of by-products (pulp, olive kernels, skin, and water) that are potential environmental pollutants due to their high organic load [3]; approximately 800 g of olive cake is obtained from each kg of olives [4]. Incorporation of these by-products into the diets of livestock might mitigate the environmental burden induced by their disposal and minimize the costs related to waste management and animal feeding, since animals become less dependent on conventional feeds such as cereal grains that can be consumed by humans [5]. In addition, their application according to bioeconomy principles enables the sustainable use of high-added value ingredients—nutraceuticals—inside the food chain, protects natural resources, and mitigates climate changes [6].

The phenolic compounds of olive products have been associated with improved cardiovascular health, low cholesterol levels, and increased longevity. A variety of substances with proven antioxidant and radical scavenging activity, such as hydroxytyrosol, oleuropein, tyrosol, caffeic acid, p-cumaric acid, verbascoside, and elenolic acid, is also contained in olive cake [7]. The direct inclusion of olive by-products in pasta and baked products improves not only their antioxidant capacity, but also their sensory attributes and shelf-life, due to their content in polyunsaturated fatty acids, phenolic compounds, and dietary fiber [8,9].

Furthermore, addition of olive by-product (OB) in pig diets could serve as a feasible approach to reducing production costs, especially in the Mediterranean region, while the quality and antioxidant capacity of the derived meat is maintained [10], as indicated by the reduction in the levels of saturated fatty acids (SFAs) and the increase in that of unsaturated fatty acids (MUFAs and PUFAs) [6,11,12]. In broilers, meat MUFAs levels are increased [13] and growth parameters [14] and carcass traits [15] are improved as an effect of OB dietary inclusion. Similar findings have been shown in laying hens, since levels of cholesterol and SFAs are reduced and those of MUFAs and PUFAs are increased in egg yolk after OB dietary supplementation [16,17], without negative effects on productive performance at a level of up to 9% [18] or 16% [16].

The market continuously seeks alternative ways of improving the health benefits and technological properties of dairy and meat products derived from ruminants. The World Health Organization [19] suggested a reduction in the intake of SFAs due to their hypercholesterolemic and thrombogenic effects that are correlated with an increased risk for cardiovascular diseases. In this framework, researchers have focused their attention on reducing SFAs content and improving nutritional properties of milk and meat through the enrichment of diets with agro-industrial by-products rich in unsaturated fatty acids, such as the olive cake.

## 2. Olive By-Products as Non-Conventional Feed Resources

Upon oil extraction, two fractions are generated: a solid residue that is generally known as crude olive cake (OC) or olive pomace and a liquid one that refers to olive mill wastewater. OC is the mixture of the olive kernel shell (or stone or pit) crushed into fragments, the skin, and the crushed grape pulp. OC chemical composition greatly varies due to the proportion of the aforementioned solid components but also the year, the geographic origin, the olive variety, culture conditions, and possible soil contamination. It can be further categorized to crude or extracted (or exhausted or defatted or depleted) OC based on residual oil content, to fresh or dry OC based on its moisture levels and to partly destoned or crude OC based on the stone removal or not [5,6]. For instance, the crude OC obtained by mechanical extraction contains residual oil and stones. The exhausted olive cake is the residue obtained after oil extraction from the crude olive cake by a solvent, usually hexane, and the partly destoned olive cake is the result of partly separating the stone (or kernel shell) from the pulp by screening or ventilation [20]. OC could be obtained according to the applied method of oil centrifugation process (three or two phase OC). The latter one, of two phase extraction, is a more efficient and environmentally friendly procedure, since the production of olive mill wastewater is minimized. Furthermore, other by-products of olive oil extraction are the olive pulp (OP), obtained when stones are completely separated from OC during oil extraction, and the olive leaves (OL), which refers to a mixture of leaves and branches from the pruning of olive trees and the cleaning of olives prior to oil extraction [5,6].

OB are the most abundant agro-industrial by-products in the Mediterranean basin and could be applied in animal and especially ruminant diets as fresh, ensiled, dried, or a component of concentrated pellets and multi-nutrient feed blocks. Their nutritional value (fiber content, crude protein, and oil) varies according to the cultivation conditions (geographic origin, year, season, etc.), the method of oil extraction (three or two phase centrifugation), form (crude, exhausted, partly-destoned, etc.), the preservation methods (drying, ensiling, etc.), and the storage conditions and time [5,21,22] (Table 1). OC digestibility is variable, depending on its type. However, apparent digestibility of organic matter and crude protein is low (0.20–0.50) due to a low nitrogen solubility and high levels of acid detergent insoluble nitrogen (70–75% of total nitrogen), while ether extract is highly digested (0.60–0.90), regardless of type of olive cake and processing method [5].

Olive by-products have traditionally been used by farmers in Mediterranean areas, but there are some limitations regarding their use as non-conventional feed resources for livestock [6]. These confinements are related to the fact that they contain low available protein content (6.6–9.9%), high ether extract (10–30%), neutral detergent fiber (23–73%) (NDF), and acid detergent lignin (12–37%) (ADL) and compounds such as phytic acid, polyphenols, and tannins that inhibit rumen cellulolytic activity and negatively affect OB palatability and digestibility [5,21,30]. Among them, the high lignin content constitutes the main obstacle towards better utilization, while the tannin content is less than 1% of DM [20]. Moreover, although they are considered as a good source of energy, this high energy content may reduce the animals’ total feed intake [6]. Additionally, they contain high fat levels, making them a good supplement for a balanced diet, but fat-rich by-products should be limited to a certain percentage (10% of total diet at most, although 5% is usually recommended). As a result, participation of olive by-products in the diets of both monogastrics and ruminants is limited (5–15%) due to high content of fiber and fat, respectively [6,31]. Nasopoulou and Zabetakis [32] concluded that a moderate intake of OB does not affect growth and improves the fatty acid profile of animal products by reducing saturated and increasing unsaturated ones in both meat and milk.

Appropriate methods of collection, transportation, and processing should be implemented in order to reduce the costs and improve the nutritive value of olive cake [33]; for example, ensiling is a low cost method of preserving olive cake without the need of additives or the cost of drying/pelleting [34,35,36]. There are several chemical, physical, or biological procedures that can be used to increase the protein content and minimize the anti-nutritional factors (phytic acid, polyphenols, and tannins) in olive cake. In general, destoning of olive cake improves its DM, NDF, crude protein digestibility, and nutritive value [37]. Ammonia or soda treatment and addition of molasses appeared to improve palatability and nutritive value of olive cake [38,39]. At the same time, the palatability of ensiled olive cake is very high, as observed in several studies in ruminants. High intake of olive cake has been reported in ruminants [40], whereas other reports shows that heifers can consume higher amounts of ensiled olive cake compared to lams and kids (heifers 58, lambs 34, kids 28 g/kg of W^0.75^) [41]. Moreover, ensiling with urea (4–5%) increased crude protein level of olive cake, but its digestibility was not improved [42,43]. On the other hand, solid-state fermentation using selected filamentous fungi is a promising biological technique that improves olive cake nutritional value and is characterized by its low cost, simplicity, and efficiency. At the same time, a more stable product is obtained, the requirements for energy are less, and smaller levels of effluents are produced compared to submerged fermentation systems [44].

## 3. Effects of Olive By-Products Dietary Supplementation in Ruminants

### 3.1. Effects of Olive By-Products Dietary Supplementation on Rumen Microbiota and Fermentation Characteristics

Olive by-products generally have low digestibility and palatability, and their high polyphenol content (particularly tannins) could decrease protein availability and microbial protein synthesis in ruminants due to the inhibitory action of polyphenols on the extracellular enzymes secreted by the ruminal microflora [45,46]. In detail, the inclusion of stoned olive cake (SOC) into the diet inhibited in vitro rumen biohydrogenation of C18 unsaturated fatty acids, resulting in a decrease in the stearic acid and an increase in vaccenic acid concentration, a fact that is possibly associated with differences in the microbial populations and activities; depression in the populations of *Butyrivibrio proteoclasticus, Neisseria weaveri* and *Ruminobacter amylophilus* [45]. Moreover, the inclusion of crude two-stage olive cake (10–12%) in feed blocks increased vaccenic acid production, since it is rich in oleic and linoleic fatty acids. The isomerization of linoleic acid as well as the desaturation of vaccenic acid in both rumen and mammary gland led to goat milk with high levels of rumenic acid and total conjugated linoleic acid (CLA) [47]. Additionally, the replacement of forage by crude olive cake at the level of 33% (or 16.6% of the total diet) in dairy sheep did not affect volatile fatty acids, ammonia production, microbial growth, bacterial diversity, protozoal, fungi, or archaea abundance, although pH and butyrate proportions were increased [48]. The inclusion of olive cake obtained with a two-stage (135 g/kg DM) or three-stage (112.5 g/kg DM) olive milling in the ewe diets indicated increased contents of α-linolenic and rumenic acids in rumen liquor (RL), respectively, while there was no diet effect on the overall composition of the RL microbiota among treatments [49]. However, in the same study, significant differences were observed for six bacterial taxa between control and treated groups. More specifically, the RL microbiota of animals fed the olive cake diets showed reduced concentration of *Anaerovibrio* genus, a result that could lead to a reduction in lipolysis, and thereby lowering the amount of PUFA that are available for biohydrogenation [49].

Incorporation of second-extraction pitted and dehydrated olive cake into the diets of Friesian steers at the level of 10–20% (on DM basis) did not influence rumen fermentation variables (pH, concentrations of ammonia and volatile fatty acids (VFA), and molar proportions of the different acids) [50]. In small ruminants, the inclusion of two-stage dried olive cake in the diet resulted in an increase of condensed tannins. Ruminal VFA concentration in goats and wethers increased, and ammonia concentration decreased. The inclusion of two-stage dried olive cake decreased urinary allantoin excretion only in wethers, indicating a greater sensitivity of wethers than of goats to olive cake condensed tannins [46] and a higher degradative efficiency of rumen microorganisms with proteolytic and cellulolytic activity in goats than in sheep [31]. Ruminal fermentation parameters (pH, VFA levels, and methane emissions) and nutrients utilization were also not disturbed in dairy goats after the partial replacement of the 20% forage by crude olive cake silages supplemented with sunflower oil at the level of 2% [51] or after the partial replacement of concentrates with a mixture of corn dried distillers’ grains containing 18% solubles, 18% dry citrus pulp, and 8% exhausted olive cake (as-fed basis) [52]. The discrepancy observed in the literature is also possibly related to the different olive varieties, different oil extraction procedures used, and mainly due to the interaction of olive by-products with other dietary components.

### 3.2. Effects of Olive By-Products Dietary Supplementation on Milk Products

#### 3.2.1. Dairy Cows, Buffaloes, and Camels

The partial replacement of roughage (8%) and concentrate (5%) in the total mixed ration by dried olive cake (DOC) [53] or its inclusion at the level of 5.6% (on DM basis) [54] did not affect milk yield and composition in dairy cows. Dried, partially destoned, olive cake dietary supplementation of dairy cows at the level of 15% (of DM) generally did not affect milk and cheese yield and chemical profile, but the nutritional properties of the derived cheese were also improved, since an increase in MUFAs and PUFAs and a decrease in SFAs were also observed. Moreover, atherogenic and thrombogenic indices were reduced, and oleic and CLA contents were increased as a result of the decreased biohydrogenation rate of oleic and linoleic intermediate by *Butyrivibrio* genus and *B. proteoclasticus* [55]. In buffaloes, no effect of DOC on milk yield, composition, and coagulation parameters was observed, whereas the oxidative stability and the dietetic—nutritional characteristics of the milk (increased MUFAs, PUFAs, unsaturated/saturated (UFA/SFA) ratio and decreased atherogenic and thrombogenic indices) were improved as an effect of tocopherols, retinol, and hydroxytyrosol that are presented in DOC [56].

Dietary supplementation with DOC at the level of 10% (on DM basis) did not influence milk yield and composition in dairy cows, with the exception of milk protein content that was increased [57]. An increase in MUFAs and a decrease in SFAs of milk and cheese were observed, whereas no effect of DOC on PUFAs was found. In detail, DOC dietary inclusion reduced palmitic acid and atherogenic and thrombogenic indices, while increasing oleic, vaccenic, stearic, and CLA in both milk and cheese of dairy cows [57]. Crude olive cake as a replacer of barley in the camel diets did not also affect milk yield, fat, or protein content but increased medium-chain fatty acids levels [58].

At the same time, the substitution of forages with 10% (DM) of ensiled olive cake had no effect on bovine milk yield and composition apart from an increase in milk fat yield. At the same time, a significant reduction in the content of SFAs and the atherogenic index was reported, whereas increased levels of long-chain and monounsaturated fatty acids as well as individual fatty acids like stearic, oleic, the sum of C18:1 trans-10 and trans-11 acids, and CLA (cis-9, trans-11 C18:2) were observed in milk and Halloumi cheese [36]. Moreover, Chaves et al. [59] concluded that inclusion of olive cake, conserved as silage, as a replacer of corn silage in the diet of lactating cows up to 15% (dry basis) does not alter milk production or its composition and feed efficiency.

#### 3.2.2. Dairy Ewes and Goats

In dairy ewes, MUFAs, oleic acid, n-6/n-3 PUFAs, and UFA/SFA ratio were increased without negative effects on chemical composition and clotting properties after the incorporation of olive cake at the level of 10–25%, leading to the improvement of the dietetic-nutritional characteristics of milk and cheese and the decrease of the atherogenic and thrombogenic indices [60,61]. Other researchers that used the same levels of olive cake (20% on DM basis) did not observe a significant effect on milk yield and composition in Awassi ewes [62]. Additionally, no diet effect on milk yield and composition was observed in a recent study conducted on ewes after the inclusion of olive cake produced with a two (135 g/kg DM) or three (112.5 g/kg DM) stage milling process, while an enrichment of milk fat with α-linolenic and oleic acids was reported in both olive cake supplemented groups [49]. On the other hand, the partial replacement of conventional concentrates by a mixture containing by-products like exhausted olive cake (80 g/kg as fed-basis) significantly increased milk protein content in dairy goats and improved the quality of goat milk by significantly increasing the levels of PUFAs, CLA, and linoleic acid, decreasing at the same time those of SFAs and the n-3 to n-6 ratio [52]. The inclusion of olive cake at a higher level (30% on DM basis) did not influence milk yield and composition, while MUFAs content in milk and the derived yoghurt and cheese was improved in Awassi dairy ewes [63]. However, the same authors in a recent experiment found that the replacement of forage and concentrate at the level of 30% by olive cake reduced milk yield (−10%) and protein and increased MUFAs content in ovine milk and attributed the observed discrepancies in the increased sample size of the latter study [64].

No significant effects on milk yield and composition of dairy ewes were demonstrated after the application of olive cake as a silage (fat: 115 g/kg DM) at the level of 100 g/kg DM (as a replacement for equivalent amounts of grass hay) [65]. The strategy of partial replacement of conventional forage by ensiled olive cake (with sunflower oil) at the level of 200 g/kg DM is also a valuable nutritional strategy in dairy goats, since it improves animal energy balance and microbial protein synthesis while ruminal fermentation, nutrients utilization, milk yield, and composition are not compromised [51]. Partial replacement of conventional roughages by ensiled crude olive cake (50%) did not affect milk yield in Chios ewes and Damascus goats and significantly increased milk fat content only in dairy ewes [34]. Moreover, the substitution of forages with 72 or 142 g/kg DM of ensiled olive cake reduced SFAs and increased the unsaturated and monounsaturated lipids as well as individual fatty acids like rumenic and linoleic acids in milk fat of Chios ewes with positive effects on human health, while the cholesterol content was not affected [36]. The application of an ensiled-mixture consisting of crude olive cake, orange pulp, and wheat straw increased MUFAs levels, UFA/SFA, and n-6/n-3 PUFAs ratio in milk of Comisana ewes [66].

Alloueedat et al. [67] showed that a mix of alternative feeds including olive cake supplemented at the level of 20–40% (of DM) could be used in ewe diets to mitigate production cost without negatively affecting intake, milk yield and composition, digestibility, animal welfare, and health. Moreover, feed blocks that contained two-stage olive cake at the level of 10–12% could be used as an alternative to reduce half of the amount of concentrate without detrimental effects on nutrient utilization, nitrogen balance, and energy efficiency and milk composition in dairy goats. Although a decrease in milk yield was observed, milk quality was improved, since an increase in CLA content and a decrease in SFAs levels and atherogenicity index were shown [47]. The aforementioned studies regarding the effects of OB dietary supplementation on milk products of ruminants are summarized in Table 2.

### 3.3. Effects of Olive By-Products Dietary Supplementation on Growth Performance, Carcass Traits, and Meat Quality Characteristics

#### 3.3.1. Beef Cattle

Although the majority of the available literature concerning the effects of olive by-products refers to small ruminants, there are also some recent studies that deal with their effects in beef cattle. Inclusion of up to 20% second-extraction pitted and dehydrated olive cake (DM basis) in the diet did not affect growth performance (final body weight and average daily gain) and rumen parameters (pH, ammonia, and volatile fatty acids) of Friesian steers [50]. However, dried partially destoned olive cake (DPDOC) dietary supplementation of Limousin bulls at the level of 7.5–15% (of DM) increased final body weight, average daily gain, carcass traits, intramuscular fat content, and meat yellowness. Moreover, DPDOC inclusion at the level of 15% reduced meat cooking loss, shear force value, and SFAs levels and increased PUFAs and MUFAs content, UFA/SFA and n-6/n-3 PUFAs ratio, and oleic acid levels [68]. Finally, incorporation of calcium soap of olive oil into the diets of Blonde D’Aquitane steers at the level of 4.8% did not affect growth performance, carcass traits, and meat sensory attributes [69].

#### 3.3.2. Sheep

An acceptable level of performance in lambs could be assured when olive cake is used as a part of the basal diet (12.2%) [70]. At the same time, the addition of higher levels of olive cake (15%) to the concentrate had no significant effect on daily gain, feed efficiency, carcass weight, and dressing percentage of lambs [71,72]. Moreover, the supplementation of lambs fed indoors or reared on a rangeland with olive cake (280 g/day) did not affect slaughter weight, carcass traits, meat yield, and quality characteristics (apart from a decrease in pH and an increase in juiciness of meat) [73]. Kotsampasi et al. [27] reached similar conclusions after the incorporation of partly destoned exhausted olive cake into lambs’ diet (80–240 g/kg diet); no effect on growth performance, carcass weight, or intramuscular fatty acid profile was observed, whereas fat color, fat firmness wetness, and overall acceptability of carcasses were improved. Moreover, partly destoned exhausted olive cake could be used in lamb finishing rations at the level of 10–20% with no adverse effects on ADG and carcass characteristics, although final body weight and feed efficiency were negatively affected at the higher level (20%) [26]. Being a cheap by-product, olive cake can be utilized as an alternative feed source for lambs up to the level of 20–30% without having any detrimental effect on their growth performance and carcass traits [74,75,76].

Moreover, the replacement of half of dietary wheat hay with sun-dried olive cake improved weight gain and final body weight of Awassi lambs with no detrimental effects on rumen parameters, nutrients intake, or digestibility [77]. In a recent experiment of the same authors, dietary inclusion of a mix of alternative feedstuffs that contained olive cake at the level of 250 or 500 g/kg also decreased production cost with no effect on feed conversion ratio, carcass traits, or meat quality characteristics of lambs. However, at the level of 500 g/kg, nutrients intake, digestibility, and lambs’ performance were negatively affected [78]. Farmers could reduce the amount of concentrate used for lambs up to 75% by using olive cake-based feed blocks (44%) as alternative supplements, since no negative effects on lamb performance are observed [79]. Taheri et al. [80] reached a similar inclusion after the inclusion of ensiled olive pulp at the level of 10–30% into the diets of ram lambs; ADG, FCR, and most carcass traits including major cuts were not adversely affected.

The replacement of the diet at the level of 44% by a mixture of agro-industrial by-products that contained exhausted olive cake did not change growth performance, pH, chemical composition, color, or texture parameters of lamb meat but increased its shelf-life (reduced lipid oxidation levels) and improved its fatty acid profile (decrease of SFAs and increase of PUFAs content) [81,82]. Furthermore, stoned olive cake dietary supplementation (35%) improved the oxidative stability of lamb meat and its combination with linseed (17% and 10%, respectively) improved the fatty acid composition of meat without compromising oxidative stability [83], with no effect on feed intake, growth performance, or carcass weight [84]. Reduced SFAs levels and increased n−6/n−3 PUFAs ratio were observed in the meat of lamb that were ad libitum fed with a silage based on olive cake and cactus pads [85]. Furthermore, polypohenols extracted from olive mill wastewaters have positive effects on kid meat, improving fatty acid profile (oleic and CLA content) and oxidative stability, suggesting the utilization of this byproduct as a source of polyphenols although this has not been investigated in any extent [86]. All the mentioned literature related to the effects of OB dietary supplementation on growth performance, carcass traits, and meat properties of lambs is summarized in Table 3.

## 4. Conclusions

According to the literature, it is clearly demonstrated that the incorporation of olive by-products (OB) into the diet of livestock and especially that of ruminants could serve as an advantageous strategy in the Mediterranean areas, allowing exploitation of an important costless agro-industrial by-product. Several methods of processing could be applied in order to improve OB nutritive value, with ensiling appearing as the most cost-effective. Nevertheless, the OB can safely be included up to 15–20% on DM basis without negative effects on ruminant digestion, given the variability of other diet components as indicated in several studies. Furthermore, nutritional properties of milk and meat are improved through OB dietary supplementation, without negative effects on growth performance and productivity. In detail, increased MUFAs and reduced SFAs levels are observed that are correlated with diminished hypercholesterolemic and thrombogenic effects, leading to the fortification of human health. In conclusion, OB utilization in ruminants’ diets reduces production costs and mitigates environmental burden, while an improvement in the nutritional value of the derived products is observed.

## Figures and Tables

**Table 1:** Composition of the different olive by-products for feeding trials.

Olive by-Product	Dry Matter (%)	Ether Extract (%)	Crude Protein (%)	NDF (%)	SFAs (%)	MUFAs (%)	PUFAs (%)	Total Phenols (%)	References
Olive cake (OC)	85–91	4–9	8–11	60–66	18–25	68–70	7–12	14	[5,23,24,25]
Partly destoned OC	89–92	1–5	3–13	54–68	47	45	8	0.65 mg GAE */g	[26,27]
Olive mill wastewater	-	0.2–1	-	-	-	-	-	0–1.2	[28]
Olive leaves (OL)	95	3–4	10–13	30–40	40	22	38	25	[5,23,29]

* gallic acid equivalent.

**Table 2:** The effects of olive by-products dietary supplementation on milk products of ruminants.

Olive By-Product	Level	Animal	Effects	Reference
PDOC	200 g/kg	ewes	No effect on clotting properties. Increase of MUFA, PUFA, n−6/n−3 ratio, UFA/SFA of milk	[60]
PDOC	98–244 g/kg	ewes	Increase of MUFA, PUFA, n−6/n−3 ratio, UFA/SFA of milk and cheese	[61]
Dried OC	300 g/kg	ewes	No effect on milk yield and composition. Increase of MUFA in milk, cheese, and yogurt	[63]
Dried OC (as a replacer of forages and concentrates)	30%	ewes	Increase of MUFA. Decrease of milk yield and protein yield	[64]
OC	20%	ewes	No effect on milk yield and composition	[62]
OCS	100 g/kg	ewes	No effect on milk yield and composition	[65]
OCS with sunflower oil	200 g/kg	goats	No effect on milk yield, milk composition, ruminal fermentation, and nutrients utilisation. Increase of energy balance and microbial protein synthesis	[51]
OCFB	10–12%	goats	No effect on nutrient utilisation, nitrogen balance, energy efficiency, and milk composition. Decrease of milk yield. Increase of CLA content. Decrease of SFA and atherogenic index	[47]
OCS (as a replacer of forages	50%	Ewes and goats	No effect of ewe and goat milk yield and composition apart from an increase in fat content of ewe milk	[34]
Ensiled mixture containing OC	30%	ewes	Increase of MUFA, n−6/n−3 PUFA, and UFA/SFA	[66]
OC (as part of a mix of alternative feedstuffs—AF)	20–40% of AF	ewes	No effect on feed intake, milk yield and composition, digestibility, animal welfare, and health	[67]
OCS	72–142 g/kg	ewes	No effect on the cholesterol content of milk. A decrease of SFAs and an increase of UFAs, MUFAs, CLA, and linoleic acid	[35]
Two-stage and three-stage OC	112.5–135 g/kg	ewes	No effect on milk yield and composition. Increase of α-linolenic and oleic acids in milk	[49]
EOC (as part of a mix of byproducts replacing concentrates)	80 g/kg	goats	No effect on milk yield and composition except for an increase in protein content. No effect on nutrient apparent digestibility, urine N excretion, N utilization. A decrease on SFAs and n−3/n−6. An increase of PUFAs, CLA, and linoleic acid.	[52]
DSOC (as a replacer of forages and concentrates)	13%	cows	No effect on milk yield and composition	[53]
DSOC	5.6%	cows	No effect on milk yield and composition	[54]
DOC	10%	cows	No effect on milk yield and composition apart from an increase in protein content. Increase of MUFA, oleic and vaccenic acids, C-18 FA, and CLA of milk and cheese	[57]
OCS	10%	cows	No effect on milk yield and composition apart from an increase in fat yield. Increase of MUFA, oleic acid and CLA of both milk and Hamoumi cheese. Decrease of SFA and atherogenic index of milk and Hamoumi cheese	[36]
OCS	15%	cows	No effect on milk yield, milk composition, and feed efficiency	[59]
DPDOC	15%	cows	No effect on yield of milk and cheese and chemical profile of cheese. Increase of MUFA, PUFA, oleic acid, and CLA. Decrease of SFA, atherogenic, and thrombogenic indices of cheese	[55]
DSOC (as a replacer of concentrates)	15.50 %	buffaloes	No effect on milk yield, milk composition, and coagulation parameters	[56]
Crude OC (as a replacer of forages)	3 kg	camels	No effect on milk yield and composition. Increase of MUFA	[58]

CLA: conjugated linoleic acid, DOC: dried olive cake, MUFAS: monounsaturated fatty acids, OCFB: feed blocks that contained olive cake, OCS: olive cake silage, PDOC: partly destoned olive cake, DSOC: dried stoned olive cake, DOC: dried olive cake, DPDOC: dried partially destoned olive cake, EOC: exhausted olive cake; PUFAs: polyunsaturated fatty acids, SFAs: saturated fatty acids, UFAs: unsaturated fatty acids.

**Table 3:** The effects of olive by-products dietary supplementation on growth performance, carcass traits, and meat properties of lambs.

Olive By-Product	Level	Effects	Reference
COC	280 g/kg	No effect on slaughter weight, ADG, carcass traits, meat yield, and quality parameters (CL, color), apart from a reduction in meat pH and an increase in meat juiciness.	[73]
DOC	100 g/kg	Increase of GR and FBW. No effect on carcass traits	[70]
DOC	300 g/kg	Reduction of ADG, FBW, carcass weight, and dressing percentage	[71]
DOC	150 g/kg	No effect on growth performance (FBW, ADG, and FCR),	[72]
DOC (as part of a mix of alternative feedstuffs—AF)	250 g/kg AF	No effect on growth performance (FBW, ADG, FCR), carcass traits and meat quality characteristics (pH, CL, WHC, SF, and color lightness and yellowness). A decrease of color redness was observed.	[78]
EOC	80 g/kg	No effect on meat pH, color, SF, and chemical composition. Increase of PUFAs content and n-6/n-3 ratio and reduction of SFAs and TBARS levels	[81]
FOW	50–150 g/kg	No effect on growth performance (FBW, ADG, FCR) and carcass traits	[76]
OCFB	440 g/kg	No effect on growth performance	[79]
OCS	240 g/kg	Reduction of FBW and ADG. No effect on FCR and carcass traits	[87]
OCS	300 g/kg	No effect on ADG, FCR, carcass characteristics, and major cuts, apart from a reduction in carcass dressing percentage	[80]
PDEOC	80–240 g/kg	No effect on growth performance (FBW, ADG, FCR), carcass traits, and meat chemical composition	[27]
PDEOC	100–200 g/kg	No effect on ADG and carcass major cuts. Reduction of hot carcass dressing percentage	[26]
PDOC	100–300 g/kg	No effect on growth performance (FBW, ADG, FCR) and carcass traits	[75]
SOC	350 g/kg	No effect on meat fatty acid profile. Improvement of meat oxidative stability and increase of tocopherols content	[83]
SOC	350 g/kg	No effect on growth performance (FBW, ADG), carcass traits, and meat fatty acid profile	[84]
UTOC	100–300 g/kg	No effect on FBW, ADG, and FCR	[43]

ADG: average daily gain, CL: cooking loss, COC: crude olive cake, DOC: dried olive cake, EOC: exhausted olive cake, FBW: final body weight, FCR: feed conversion rate, FOW: fermented olive wastes, GR: growth rate, OCS: olive cake silage, OCFB: feed blocks that contained olive cake, PDEOC: partly destoned exhausted olive cake, PDOC: partly destoned olive cake, PUFAs: polyunsaturated fatty acids, SF: shear force, SFAs: saturated fatty acids, SOC: stoned olive cake, TBARS: thiobarbituric acid reactive substances, UTOC: urea treated olive cake, WHC: water holding capacity.

## Data Availability

Data sharing not applicable.
